# Protective Effects of Resveratrol and Apigenin Dietary Supplementation on Serum Antioxidative Parameters and mRNAs Expression in the Small Intestines of Diquat-Challenged Pullets

**DOI:** 10.3389/fvets.2022.850769

**Published:** 2022-05-30

**Authors:** Ning Zhou, Yong Tian, Wenchao Liu, Bingjiang Tu, Wenwu Xu, Tiantian Gu, Kang Zou, Lizhi Lu

**Affiliations:** ^1^College of Animal Science and Technology, Nanjing Agricultural University, Nanjing, China; ^2^State Key Laboratory for Managing Biotic and Chemical Threats to the Quality and Safety of Agro-products, Institute of Animal Science and Veterinary, Zhejiang Academy of Agricultural Science, Hangzhou, China; ^3^Huzhou Lvchang Ecoagriculture Co., Ltd., Huzhou, China; ^4^Animal Disease Prevention and Control Center, Huzhou Wuxing District Agricultural and Rural Bureau, Huzhou, China

**Keywords:** resveratrol, apigenin, diquat, oxidative stress, mRNA expression, small intestine, pullets

## Abstract

Poultry as a large-scale intensive farming is vulnerable to oxidative stress. Resveratrol and apigenin are recognized to have many beneficial bioactive functions. This study tested the hypothesis that dietary resveratrol and apigenin supplementation alleviates oxidative stress in the small intestine of diquat-challenged pullets. A total of 200 healthy pullets were randomly divided into four treatment groups: control group fed with a basal diet (CON), diquat group fed with a basal diet (DIQ), resveratrol group fed with a basal diet containing 500 mg/kg resveratrol (RES), and an apigenin group fed with a basal diet containing 500 mg/kg apigenin (API) and injected intraperitoneally with either 1 ml of saline (CON) or 8 mg/kg body weight of diquat (DIQ, RES, and API) to induce oxidative stress. The day of the injection was considered as day 0. The results indicated that resveratrol and apigenin were able to decrease the malondialdehyde (MDA) level and upregulate total antioxidant capacity (T-AOC), superoxide dismutase (SOD), and glutathione peroxidase (GSH-PX) levels in serum on day 1 and 10 after being diquat-challenged. In addition, resveratrol increased mRNA expression of *NQO1* (NAD(P)H dehydrogenase quinone 1) and *HO-1* (heme oxygenase-1) in ileum and jejunum on day 10, while apigenin upregulated nuclear factor erythroid 2-related factor 2 (*NRF2*), *NQO1*, and *HO-1* in ileum and jejunum on day 10. Both resveratrol and apigenin increased the mRNA expression of *CLAUDIN-1* in ileum and jejunum on day 1 and that of *ZO-1* (zonula occludens-1) in ileum on day 10 post-diquat-injection. These findings indicate that dietary supplementation with resveratrol and apigenin attenuates oxidative stress involving NRF2 signaling pathways in diquat-challenged pullets to some extent. These observations are valuable for the chicken industry and resveratrol and apigenin applications in animal husbandry.

## Graphical Abstract

The process of resveratrol and apigenin to alleviate diquat-induced oxidative stress in epithelial cells. 
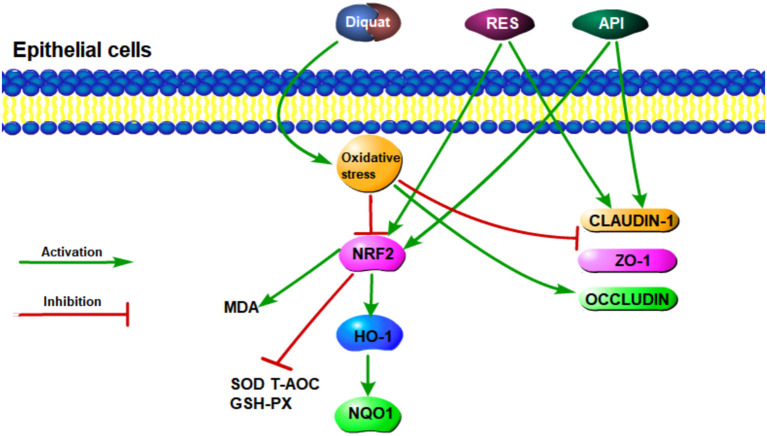

## Introduction

Intensive modern farming significantly boosts livestock productivity and economic benefit and increases the risk of animals' exposure to oxidative stress (1). Various factors such as physics/chemistry, nutrition, temperature, and local environment can induce oxidative stress, which represents the imbalance between reactive oxygen species (ROS) production and defense responses of animals (2). When this balance is disrupted, increased ROS will further alter antioxidant defense capacities, including changes in malondialdehyde (MDA), superoxide dismutase (SOD), catalase (CAT), total antioxidant capacity (T-AOC), and glutathione peroxidase (GSH-PX) levels (3–5). Excessive ROS generation could damage cellular macromolecules, DNA, and proteins to impair cellular functions; thus, affecting animal survival and resulting in economic losses in animal farming (6). As a natural barrier between the internal and external environments of pullets, the intestine is susceptible to oxidative stress. Therefore, it is imperative to establish appropriate nutrition strategies to decrease the risk of intestinal oxidative damage in pullets.

Recent studies have shown that oxidative stress can disturb cellular functions by influencing transcription factors and the redox-sensitive signaling pathway, nuclear factor erythroid 2-related factor 2 (NRF2), NAD(P)H dehydrogenase quinone 1 (NQO1), and heme oxygenase-1 (HO-1) regarded as transcription factors exerting critical regulatory effects on the oxidative status via induced expression of the antioxidant and phase-2 detoxifying enzymes (7–9). Normal intestinal functioning depends on the initiation and conservation of a mucosal barrier, and this intestinal mucosal barrier is indispensable for preventing intestinal injury due to certain microorganisms or undesirable substances (10). Several studies have demonstrated that oxidative stress and disruption of cellular redox status impaired intestinal functioning and intestinal turnover (11–13). The formation of tight junctions creates the significant components of the intestinal barrier: OCCLUDIN is the first tight junction protein to be identified, whereas CLAUDINS and ZO-1 (zonula occludens-1) are the main proteins contributing to the physiological and structural paracellular barrier function (14).

Diquat, a bipyridyl herbicide, is able to convert molecular oxygen into superoxide anion radical and stimulate cellular production of free radical species via cyclic reduction-oxidation processes and is often used to induce oxidative stress (15–19). Diquat-challenging oxidative stress has been reported to affect intestinal morphology and disrupt intestinal function (17–20). The maintenance of the intestinal epithelial redox environment is essential for the activities of pivotal physiological processes, such as digestion and absorption, cell proliferation and apoptosis, and immune response (21). Many factors lead to oxidative stress (22, 23) and ultimately affect intestinal health. Resveratrol is a nutraceutical that has garnered much attention because of its antioxidant and anti-apoptosis potential, being a phytoalexin polyphenolic compound found in many kinds of plants, such as grapes and peanuts, among others. Evidence suggest that that dietary resveratrol supplementation enhances the antioxidant status of the animal body and/or animal products (24, 25). Besides this, resveratrol exerts a strong inhibitory effect on the production of ROS in many experimental systems, with anti-inflammatory, anti-senescence, and anti-obesity among its various biological functions (26, 27). For poultry, resveratrol can be used as a feed additive to improve the meat quality of broilers, an outcome that may be associated with an improved muscle antioxidative status and mitochondrial biogenesis (28, 29). Apigenin is a natural phytochemical, a flavonoid, which is present in several dietary plant foods, namely, vegetables and fruits (30). A few studies conducted in recent years have shown that apigenin is a potential antioxidant, anti-apoptosis, and anti-inflammatory agent (30–33). In this context, surprisingly, limited information is available on the effects of resveratrol and apigenin to ameliorate diquat-induced oxidative stress and intestinal barrier dysfunction in pullets. Hence, the objective of this study is to evaluate the influence of resveratrol and apigenin for mitigating oxidative stress-induced impairment of serum antioxidative capacity, intestinal morphology, and mRNA expression levels of the NRF2 pathway and tight junctions-related genes in pullets.

## Materials and Methods

### Animals, Diets, and Management

This experimental trial was conducted at Huzhou Lvchang Ecoagriculture Co., Ltd., in Zhejiang Province. A total of 200 healthy, 13-week-old Jingfen NO.1, pullets (1.17 kg ± 0.24) were recruited and randomly assigned to four dietary treatments with five replicates (*n* = 10 pullets per replicate) as follows: (1) control group (CON), pullets fed with a basal diet and injected with sterile saline; (2) diquat-challenged group (DIQ), pullets fed with a basal diet and injected with diquat; (3) resveratrol group (RES) + diquat, pullets fed with a basal diet containing 500 mg/kg resveratrol and injected with diquat; (4) apigenin group (API) + diquat, pullets fed with a basal diet containing 500 mg/kg apigenin and injected with diquat. The diquat was purchased from Shangdong Baishiwei Crop Protection Co., Ltd; the resveratrol came from Chengdu Huagao Biological Products Co. Ltd, and the apigenin was bought from Changsha Shanghe Biological Technology Co. Ltd. The supplemental level of resveratrol was based on a previous study (34). The supplemental level of apigenin is the same as that of resveratrol. All the chickens were fed different diets for 7 days and then injected intraperitoneally with either 1 ml of saline or 8 mg/kg, which was according to the previous report (19), diquat dissolved in 1 ml of saline to induce oxidative stress. Five pullets were caged in a single pen (40 × 50 × 40 cm), with 3-tiered battery cages and diet and freshwater offered *ad libitum*. The photoperiod regimen was set to 16-h-light: 8-h-dark during the experimental period. The temperature and light conditions were the same across the different treatment groups. The basal diets provided to meet the nutritional requirements of hens are shown in Table 1. According to the Local Experimental Animal Care Committee, all procedures were implemented and approved by Nanjing Agricultural University.

**Table 1:** Ingredients and nutrient composition of the basal diet.

**Ingredients**	**Content (%)**	**Nutrient levels**	**Content**
Corn	61.03	Metabolic energy^b^ (MJ/kg)	11.20
Soybean meal	32.52	Crude protein^c^, %	16.35
Wheat bran	2.00	Lysine^c^, %	0.87
Soybean oil	0.45	Cysteine + Methionine^c^, %	0.68
Vitamin-mineral premix^1^	4.00	Calcium^c^, %	3.50
Total	100	Available phosphorus^c^, %	0.37

*^a^The premix provided the following nutrients per kilogram of diet: VA, 200000 IU; VD3, 80000 IU; VE, 600 IU; VK_3_, 62 mg; VB_1_, 50 mg; VB_3_, 150 mg; VB_6_, 90 mg; VB_12_, 0.5 mg; niacin, 800 mg; pantothenic acid, 350 mg; folic acid, 30 mg; biotin, 6 mg; choline chloride, 7,800 mg; Fe, 1,500 mg; Cu, 250 mg; Mn, 65 mg; Zn, 1,900 mg; Se, 5.8 mg; I, 23 mg*.

b *Calculated by NRC (1994) nutrient requirement for pullets*.

c *Analysed content*.

### Sample Collection

Day 0 corresponded to when the intraperitoneal injection was administered; then, on day 1 and 10 of the trial, one pullet was randomly selected from each group replicate (40 pullets in total). Blood samples were drawn from the axillary vein into vacuum tubes (5 ml) containing coagulant and then centrifuged at 3,000 × *g* for 10 min, after which the ensuing serum was stored at −20°C until further analysis. The pullets were slaughtered by intracardial administration of sodium pentobarbital (30 mg/kg of body weight, Sinopharm, China) and jugular exsanguination after overnight feed deprivation according to a previous article (35). Samples of the jejunum and ileum were removed from the middle of the jejunum segment and ileum segment and then rinsed with ice-cold phosphate-buffered saline (PBS, Solarbio, China). One section of the jejunum and ileum samples were immediately frozen in liquid nitrogen, then transferred to storage at −80°C until further analysis. Other sections of intestinal samples (3 cm, taken on day 10) were fixed in 4% para form (Biosharp, Shanghai, China) and stored at room temperature for morphological analysis.

### Estimation of Serum Antioxidant Parameters

The activities of the total antioxidant capacity (T-AOC), the superoxide dismutase (SOD), the glutathione peroxidase (GSH-PX) activity, and the malondialdehyde (MDA) in the serum were determined to estimate the oxidative status of the pullets. In brief, the T-AOC level was evaluated by the FRAP method. The SOD activity was detected by the WST method. The activity of GSH-PX was determined by the 5,5'-dithiobis- (2-nitrobenzoic acid) method. The MDA level was measured by the TBA reaction method. The T-AOC (HY-60021), SOD (HY-M0001), GSH-PX (HY-60005), and MDA (HY-60003) assay kits purchased from the Beijing Sino-UK Institute of Biological Technology were used. All experiments were performed according to the manufacturer's instructions.

### Histological Assay

Small intestine samples, i.e., jejunum and ileum, were prepared for histological analysis. First, these samples in para form solution were embedded in paraffin and cut into 5-μm-thick sections. Next, hematoxylin–eosin staining was carried out using this sequence of procedures: dehydration, embedding, sectioning, and staining. Villous height and crypt depth were measured from three discontinuous sections of each sample were made for observation, and six complete, typical fields of view were selected for each sample using an optical binocular microscope (Olympus BX5; Olympus Optical Co. Ltd, Tokyo, Japan) equipped with a digital camera (Nikon Eclipse Ci-L; Nikon, Tokyo, Japan) and an image analyzer (Image-Pro Plus 6.0; Media Cybernetics, Bethesda, MD, U.S.A.) (35).

### Total RNA Isolation and Quantitative Real-Time PCR

The total RNA was extracted from different animal tissues with the E.Z.N.A total RNA Kit II (OMEGA Bio-Tek, Norcross, USA), was then stored at −80°C until the cDNA synthesis. The RNA quality was measured using a Nanodrop spectrophotometer (Thermo Fisher, Waltham, USA) at 260 and 280 nm. Approximately, 1 μg of RNA was reverse transcribed into cDNA in a total volume of 10 μl by using the HiScript RII Q RT SuperMix for qPCR (+gDNA wiper [Vazyme, R223-01, Nanjing, China]). The levels of *SOD-1, CAT, GPX-1, NRF2, NQO1, HO-1, CLAUDIN-1, OCCLUDIN*, and *ZO-1* expression were determined in jejunum and ileum samples. All primer sequences (Table 2) were designed in Primer 5.0 software. The PCR reactions were performed in a LightCycler96 (Roche, Basel, Switzerland), using the SYBR Green PCR Master Mix (Vazyme, Q711-02/03, Nanjing, China) to the manufacturer's protocol. The cycle threshold was collected from each reaction, and the relative expression level of different genes' mRNA to the β*-actin* mRNA was evaluated using the 2^−ΔΔCT^ method. β*-actin* is used as an internal control to normalize target gene transcript levels.

**Table 2:** Characteristics of the primers used for the real-time PCR analysis.

**Genes**	**Primer (from 5^**′**^to 3^**′**^)**	**Products**	**Accession**
		**size (bp)**	**number**
*SOD-1*	F: GGCAATGTGACTGCAAAGGG	133	NM_205064.1
	R: CCCCTCTACCCAGGTCATCA		
*CAT*	GGGGAGCTGTTTACTGCAAG	139	NM_001031215.2
	GGGGAGCTGTTTACTGCAAG		
*GPX-1*	F: AACCAATTCGGGCACCAG	122	HM590226
	R: CCGTTCACCTCGCACTTCTC		
*NRF2*	F: GAGCCCATGGCCTTTCCTAT	212	NM_001007858.1
	R: CACAGAGGCCCTGACTCAAA		
*NQO1*	F: TCGCCGAGCAGAAGAAGATTGAAG	192	NM_001277620.1
	R: CGGTGGTGAGTGACAGCATGG		
*HO-1*	F: AAGAGCCAGGAGAACGGTCA	121	NM_205344
	R: AAGAGCCAGGAGAACGGTCA		
*Cloudin-1*	F: GCATGGAGGATGACCAGGTGA	117	NM_001013611.2
	R: GAGCCACTCTGTTGCCATACCAT		
*Occludin*	F: GCAGATGTCCAGCGGTTACTAC	176	NM_205128.1
	R: CGAAGAAGCAGATGAGGCAGAG		
*ZO-1*	F: AAGTGTTTCGGGTTGTGGAC	160	XM_413773.4
	R: GCTGTCTTTGGAAGCGTGTA		
*β-actin*	F: CACCACAGCCGAGAGAGAAAT	135	L08165
	R: TGACCATCAGGGAGTTCATAGC		

*SOD-1, superoxide dismutase 1; CAT, catalase; GPX-1, glutathione peroxidase 1; NRF2, nuclear factor erythroid 2-related factor 2; NQO1, NAD(P)H dehydrogenase quinone 1; HO-1, heme oxygenase-1; ZO1, zonula occludens-1*.

### Statistical Analysis

This analysis was implemented in SPSS 15.0 (Statistical Product and Service Solutions, Inc., USA) and plotted to utilize GraphPad Prism 8 (GraphPad, CA, USA). One-way analysis of variance (ANOVA) followed by the least significant difference (LSD) test was used for multiple comparisons with each pullet as the experimental unit. Results were presented as means ± the standard error of the mean (SEM). *p* < 0.05 were considered statistically significant.

## Results

### Serum Antioxidative Capacity

According to the results given in Table 3, on day 1 post-injection, the serum MDA concentration of pullets was higher in the DIQ group than in CON groups injected with normal saline (*p* < 0.05). Meanwhile, the serum MDA concentration of the DIQ group supplemented with the basal diet was higher than that of samples supplemented with resveratrol and apigenin (*p* < 0.05). The diquat treatment tended to block serum SOD activity (*p* < 0.05), but resveratrol and apigenin markedly restored (*p* < 0.05) that inhibited function when compared with the DIQ group. The GSH-PX concentration significantly decreased after exposure to diquat (*p* < 0.05). In contrast, dietary supplementation with resveratrol and apigenin increased the serum GSH-PX level when compared with the DIQ group (*p* < 0.05). The serum T-AOC level was more remarkable in pullets fed a diet with 500 mg/kg of resveratrol or apigenin with the diquat treatment than that in the DIQ group. The antioxidant status of serum was examined on day 10 after the diquat injection (Table 3). Compared with the CON group, the diquat significantly increased the serum MDA concentration and decreased the concentrations of SOD, GSH-PX, and T-AOC. On the contrary, resveratrol and apigenin attenuated the enhanced MDA levels induced by diquat and augmented the SOD, GSH-PX, and T-AOC levels compared with the DIQ group.

**Table 3:** Serum antioxidative status activity of pullets fed with resveratrol and apigenin on day 1 and day 10 after their injection with diquat.

**Item**	**CON**	**DIQ**	**RES**	**API**
**Day 1**
MDA	3.69 ± 0.14^b^	4.57 ± 0.14^a^	3.16 ± 0.19^c^	3.62 ± 0.16^b^
SOD	71.72 ± 3.78^a^	53.03 ± 2.26^b^	78.38 ± 5.16^a^	72.88 ± 7.83^a^
GSH-PX	666.01 ± 37.90^c^	573.90 ± 20.27^d^	936.94 ± 30.89^a^	881.79 ± 26.16^b^
T-AOC	10.72 ± 0.46^b^	8.74 ± 0.35^c^	12.96 ± 0.56^a^	12.27 ± 0.44^a^
**Day 10**
MDA	2.88 ± 0.24^b^	3.47 ± 0.16^a^	2.93 ± 0.13^b^	2.90 ± 0.17^b^
SOD	88.29 ± 6.53^a^	71.24 ± 4.44^b^	90.29 ± 2.49^a^	88.19 ± 3.21^a^
GSH-PX	1034.91 ± 8.83^b^	930.17 ± 5.17^c^	993.77 ± 6.67^bc^	1125.66 ± 19.91^a^
T-AOC	13.52 ± 0.38^b^	11.42 ± 0.34^c^	15.09 ± 0.61^a^	15.71 ± 0.35^a^

a, b, c, d *Means without a common superscript with a row differ significantly (p < 0.05). MDA, malondialdehyde, nmol/ml; SOD, superoxide dismutase, U/ml; GSH-PX, glutathione peroxidase, U/ml; T-AOC, total antioxidant capacity, U/ml. CON, pullets fed basal diet; DIQ, diquat-injection pullets; RES, pullets fed with a basal diet containing resveratrol and injected with diquat; API, pullets fed with a basal diet containing apigenin and injected with diquat*.

### Expression of Antioxidant Enzyme Genes in Ileum Tissue and in Jejunum Tissue

**Figure 1:**
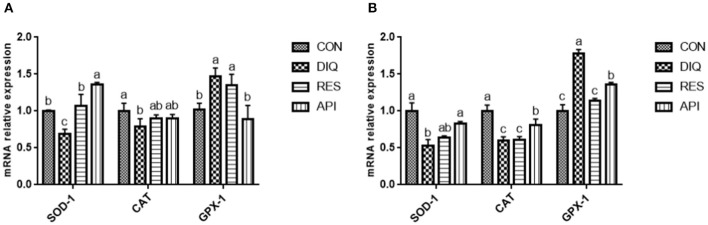
Effects of dietary resveratrol and apigenin supplementation on mRNA expression of antioxidant genes in ileum and jejunum on day 10. **(A)** Related genes expression level in the ileum of pullets. **(B)** Related genes expression level in the jejunum of pullets. *SOD-1*, superoxide dismutase 1; *CAT*, catalase; *GPX-1*, glutathione peroxidase 1. CON, pullets fed basal diet; DIQ, diquat-injection pullets; RES, pullets fed with a basal diet containing resveratrol and injected with diquat; API, pullets fed with a basal diet containing apigenin and injected with diquat. Data are means ± standard error; mean values sharing different lowercase letters differ significantly (*p* < 0.05).

As shown in Figure 1A, the expression levels of *SOD-1* and *CAT* in ileum tissue of pullets on day 10 were lower in the DIQ group than those in the CON group (*p* < 0.05). Resveratrol and apigenin supplementation elevated the expression level of *SOD-1* in ileum tissue compared to the CON group (*p* < 0.05). Moreover, on day 10, the expression levels of *SOD-1* and *CAT* in jejunum tissue of pullets were lower in the DIQ group than those in the CON group (*p* < 0.05) (Figure 1B). On the contrary, the expression levels of *SOD-1* and *CAT* in jejunum tissue were significantly increased in the API group, as compared to those in the DIQ group (*p* < 0.05). The expression level of *GPX-1* in ileum tissue and jejunum tissue was upregulated in the DIQ group than those in the CON group (*p* < 0.05), and resveratrol and apigenin supplementation could reverse in jejunum tissue (*p* < 0.05).

### Intestinal Morphology

**Figure 2:**
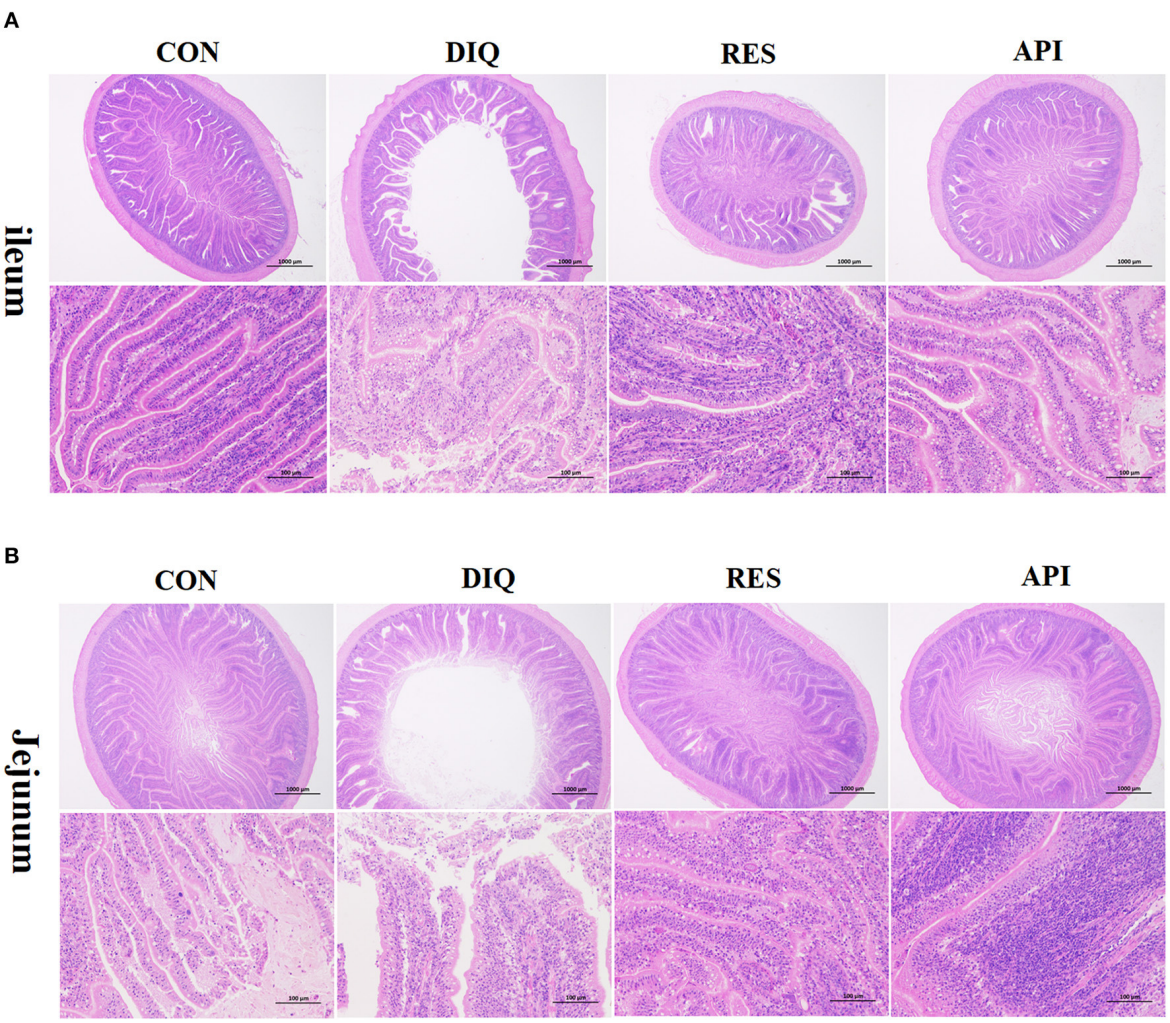
Effect of resveratrol and apigenin on intestinal morphological structure in diquat-induce oxidative stress with chickens on day 10 after injection with diquat (HE, ×20 and ×200). **(A)** The images of the ileum morphology. **(B)** The images of the jejunum morphology. CON, pullets fed basal diet; DIQ, diquat-injection pullets; RES, pullets fed with a basal diet containing resveratrol and injected with diquat; API, pullets fed with a basal diet containing apigenin and injected with diquat.

Morphology, villus height, and crypt depth of the jejunum and ileum on day 10 are shown in Figure 2 and Table 4. In the ileum tissue, the structure of the CON group gut was clear, the mucosal layer epithelium was complete, and the intestinal villi were arranged regularly. In the DIQ group, the villus height was higher (*p* < 0.05) compared with those of the CON group, whereas in both RES and API groups, the villus height of the RES and API group was higher than that of the DIQ group (*p* < 0.05). Similarly, for the morphology of jejunum tissue, compared with CON, the villus height of the jejunum was significantly decreased in DIQ (*p* < 0.05). However, the dietary supplement containing resveratrol alleviated this abnormal condition by increasing the height of the intestinal villi of pullets under stress (*p* < 0.05).

**Table 4:** Effects of resveratrol and apigenin on intestinal morphology in ileum and jejunum of pullets with injected diquat on day 10.

**Items**	**CON**	**DIQ**	**RES**	**API**
**Ileum**
Villus height (μm)	1166.82 ± 39.54^a^	743.52 ± 25.99^c^	995.66 ± 28.16^b^	956.65 ± 27.17^b^
Crypt depth (μm)	201.33 ± 12.31^a^	141.44 ± 12.96^b^	142.49 ± 3.67^b^	150.82 ± 11.53^b^
Villus heigh/crypt depth	5.90 ± 0.39^ab^	5.49 ± 0.55^b^	7.01 ± 0.29^a^	6.47 ± 0.33^ab^
**Jejunum**	
Villus height (μm)	1437.62 ± 46.19^a^	971.84 ± 18.34^c^	1111.70 ± 41.46^b^	985.95 ± 22.71^c^
Crypt depth (μm)	197.47 ± 4.28^a^	138.90 ± 7.99^b^	154.85 ± 8.11^b^	138.24 ± 4.59^b^
Villus heigh/crypt depth	7.27 ± 0.10	7.07 ± 0.27	7.21 ± 0.16	7.14 ± 0.08

a, b, c * Means without a common superscript with a row differ significantly (p < 0.05). CON, pullets fed basal diet; DIQ, diquat-injection pullets; RES, pullets fed with a basal diet containing resveratrol and injected with diquat; API, pullets fed with a basal diet containing apigenin and injected with diquat*.

### Relative Expression of the NRF2 Signaling Pathway and Tight Junction mRNAs in Ileum Tissue

**Figure 3:**
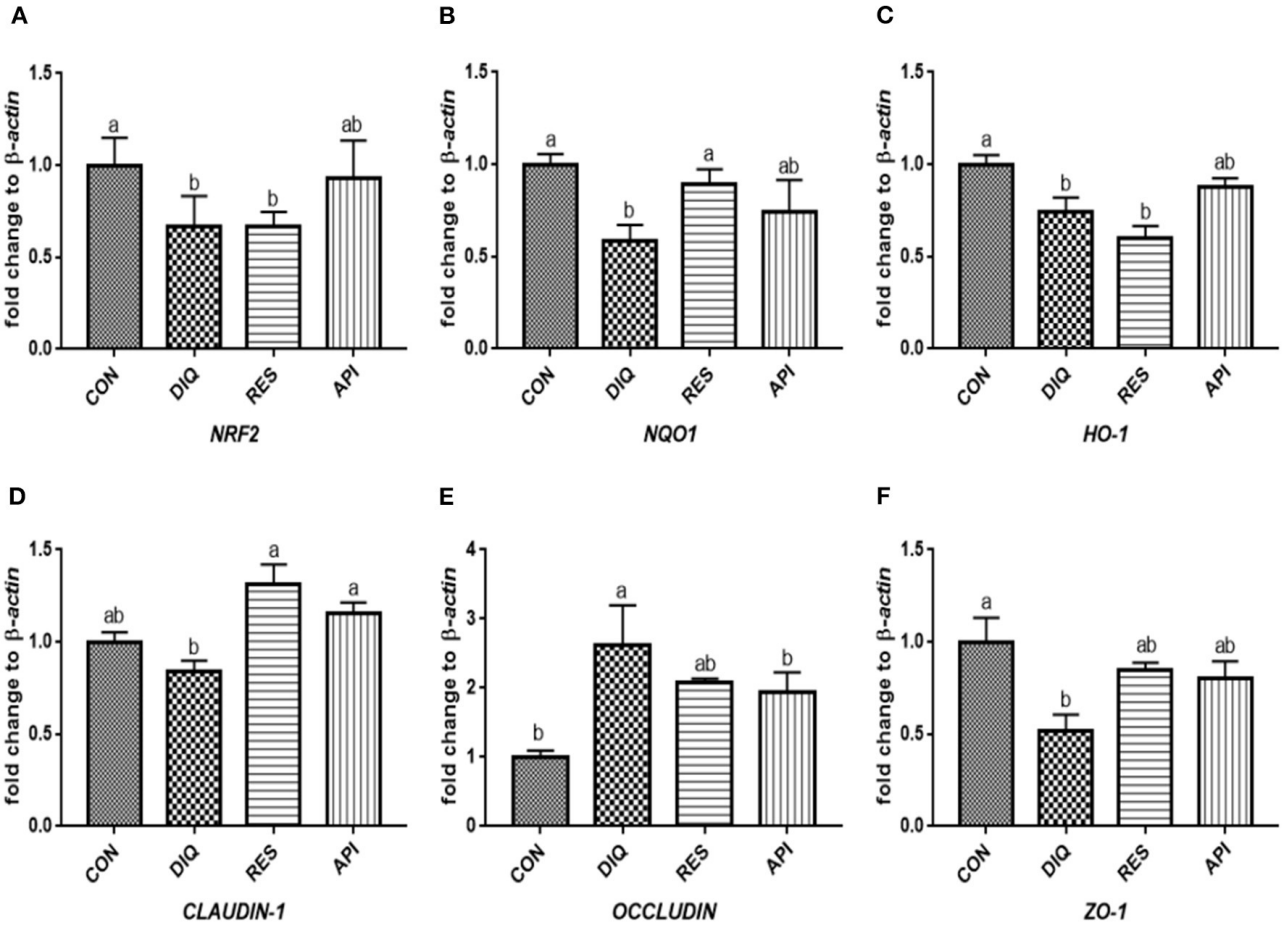
Effects of dietary resveratrol and apigenin supplementation on mRNA expression of antioxidant genes and tight junction RNAs in ileum on day 1. **(A)** The expression level of NRF2 gene in ileum from different groups. **(B)** The expression level of NQO1 gene in ileum from different groups. **(C)** The expression level of HO-1 gene in ileum from different groups. **(D)** The expression level of CLAUDIN-1 gene in ileum from different groups. **(E)** The expression level of OCCLUDIN gene in ileum from different groups. **(F)** The expression level of ZO-1 gene in ileum from different groups. NRF2, nuclear factor erythroid 2-related factor 2; NQO1, NAD (P) H dehydrogenase quinone 1; HO-1, heme oxygenase-1; ZO1, zonula occludens-1. CON, pullets fed basal diet; DIQ, diquat-injection pullets; RES, pullets fed with a basal diet containing resveratrol and injected with diquat; API, pullets fed with a basal diet containing apigenin and injected with diquat. Data are means ± standard error; mean values sharing different lowercase letters differ significantly (*p* < 0.05).

**Figure 4:**
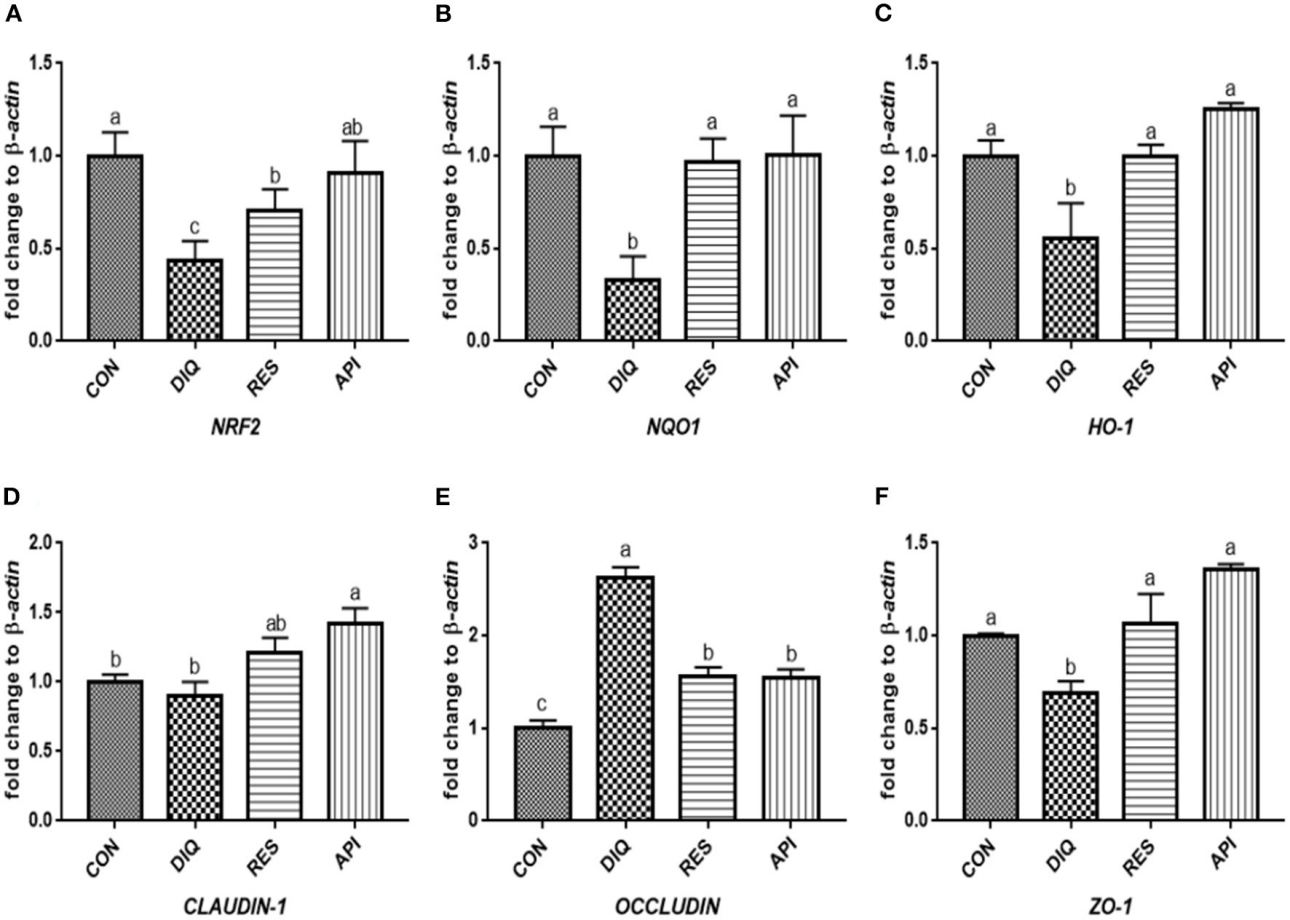
Effects of dietary resveratrol and apigenin supplementation on mRNA expression of antioxidant genes and tight junction RNAs in ileum on day 10. **(A)** The expression level of NRF2 gene in ileum from different groups. **(B)** The expression level of NQO1 gene in ileum from different groups. **(C)** The expression level of HO-1 gene in ileum from different groups. **(D)** The expression level of CLAUDIN-1 gene in ileum from different groups. **(E)** The expression level of OCCLUDIN gene in ileum from different groups. **(F)** The expression level of ZO-1 gene in ileum from different groups. NRF2, nuclear factor erythroid 2-related factor 2; NQO1, NAD (P) H dehydrogenase quinone 1; HO-1, heme oxygenase-1; ZO1, zonula occludens-1. CON, pullets fed basal diet; DIQ, diquat-injection pullets; RES, pullets fed with a basal diet containing resveratrol and injected with diquat; API, pullets fed with a basal diet containing apigenin and injected with diquat. Data are means ± standard error; mean values sharing different lowercase letters differ significantly (*p* < 0.05).

The relative expression levels of mRNAs involved in the NRF2 signaling pathway on day 1 are shown in Figures 3A–C. When compared with the saline-treated pullets, diquat-treated ones fed the basal diet had lower mRNA abundances of *NRF2, NQO1*, and *HO-1* in their ileum (*p* < 0.05). In contrast, the diquat-challenged chickens fed the diet supplemented with resveratrol and apigenin did not differ from the DIQ group in their *NRF2* and *HO-1* mRNA expression (*p* > 0.05). The levels *NRF2, NQO1*, and *HO-1* mRNA expression of the API group were similar to those of the DIQ group. The mRNA expression levels of *CLAUDIN-1, OCCLUDIN*, and *ZO-1* in the ileum are presented in Figures 3D–F. Pullets in the RES and API groups exhibited higher *CLAUDIN-1* mRNA levels in the ileum than it in the DIQ group (*p* < 0.05). In addition, both resveratrol and apigenin supplementation groups tended to undergo greater *ZO-1* mRNA expression than the DIQ group (*p* > 0.05). Concerning the mRNA expression of *OCCLUDIN*, its level significantly increased in the DIQ group compared with the CON group, but this phenomenon was partly relieved in the supplementation groups. Next, we performed qPCR to test the validity of expressed mRNA of *NRF2, NQO1, HO-1, CLAUDIN-1, OCCLUDIN*, and *ZO-1* in the ileum of the four groups on day 10 (Figure 4). Compared with the CON group, the expression levels of *NRF2, NQO1*, and *HO-1* were significantly decreased in the DIQ group but increased in the RES group and API group (*p* < 0.05). Further, the mRNA expression level of *OCCLUDIN* was downregulated, and the *ZO-1* mRNA expression level was upregulated in the RES and API groups compared with those in the DIQ group (*p* < 0.05).

### Relative Expression of the NRF2 Signaling Pathway and Tight Junction mRNAs in Jejunum Tissue

**Figure 5:**
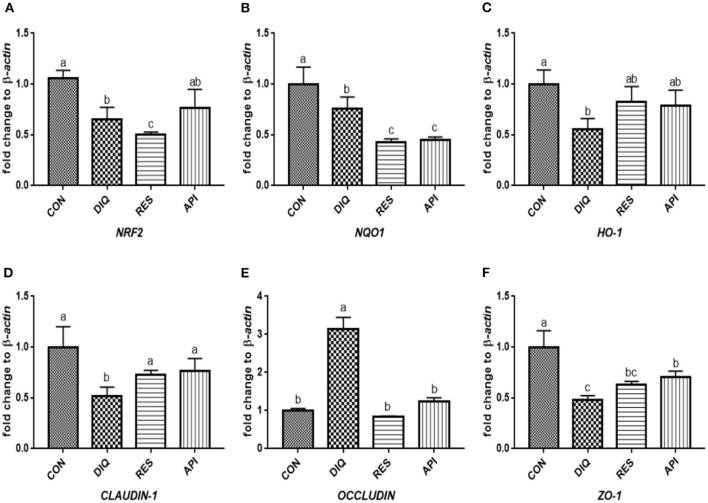
Effects of dietary resveratrol and apigenin supplementation on mRNA expression of antioxidant genes and tight junction RNAs in jejunum on day 1. **(A)** The expression level of NRF2 gene in jejunum from different groups. **(B)** The expression level of NQO1 gene in jejunum from different groups. **(C)** The expression level of HO-1 gene in jejunum from different groups. **(D)** The expression level of CLAUDIN-1 gene in jejunum from different groups. **(E)** The expression level of OCCLUDIN gene in jejunum from different groups. **(F)** The expression level of ZO-1 gene in jejunum from different groups. NRF2, nuclear factor erythroid 2-related factor 2; NQO1, NAD (P) H dehydrogenase quinone 1; HO-1, heme oxygenase-1; ZO1, zonula occludens-1. CON, pullets fed basal diet; DIQ, diquat-injection pullets; RES, pullets fed with a basal diet containing resveratrol and injected with diquat; API, pullets fed with a basal diet containing apigenin and injected with diquat. Data are means ± standard error; mean values sharing different lowercase letters differ significantly (*p* < 0.05).

**Figure 6:**
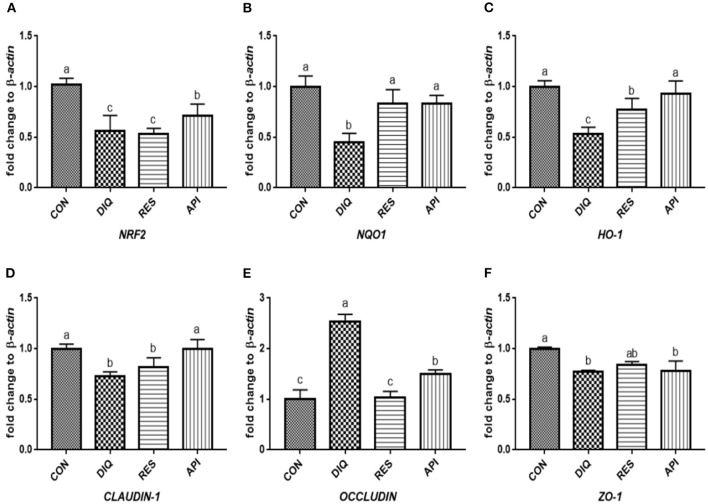
Effects of dietary resveratrol and apigenin supplementation on mRNA expression of antioxidant genes and tight junction RNAs in jejunum on day 10. **(A)** The expression level of NRF2 gene in jejunum from different groups. **(B)** The expression level of NQO1 gene in jejunum from different groups. **(C)** The expression level of HO-1 gene in jejunum from different groups. **(D)** The expression level of CLAUDIN-1 gene in jejunum from different groups. **(E)** The expression level of OCCLUDIN gene in jejunum from different groups. **(F)** The expression level of ZO-1 gene in jejunum from different groups. NRF2, nuclear factor erythroid 2-related factor 2; NQO1, NAD (P) H dehydrogenase quinone 1; HO-1, heme oxygenase-1; ZO1, zonula occludens-1. CON, pullets fed basal diet; DIQ, diquat-injection pullets; RES, pullets fed with a basal diet containing resveratrol and injected with diquat; API, pullets fed with a basal diet containing apigenin and injected with diquat. Data are means ± standard error; mean values sharing different lowercase letters differ significantly (*p* < 0.05).

The day 1 detection results (Figure 5) revealed a trend of downregulated *NRF2, NQO1*, and *HO-1* mRNA levels when exposed to diquat (*p* < 0.05). The *NRF2* and *HO-1*mRNA levels of the DIQ group were on par with those of the API group (*p* > 0.05). Compared with the CON group, the diquat injection lowered the mRNA abundance of *CLAUDIN-1* (*p* < 0.05). At the same time, resveratrol and apigenin increased the *CLAUDIN-1* gene levels (*p* < 0.05), and vice versa for the *OCCLUDIN* gene. *ZO-1* expression of the DIQ group was not significantly different from the RES group (*p* > 0.05), whereas it was significantly lower than that of the API group (*p* < 0.05). The relative mRNA expression levels of the Nrf2 signaling pathway and tight junction genes (*NRF2, NQO1, HO-1, CLAUDIN-1, OCCLUDIN*, and *ZO-1*) on day 10 are shown in Figure 6. Unlike the trends on day 1, the *NQO1* and *HO-1* mRNA levels of both RES and API groups were significantly those of the DIQ group (*p* < 0.05). *CLAUDIN-1* and *ZO-1* mRNA expression levels of the DIQ group were downregulated compared to the CON group (*p* < 0.05). Moreover, *ZO-1* mRNA expression has no significant difference in the RES or API group than in the DIQ group (*p* > 0.05).

## Discussion

In the present study, we relied on diquat to induce intestinal oxidative stress injury in pullets and examined the protective function of resveratrol and apigenin toward mitigating that process. Our results showed that the serum concentration of MDA rose after diquat injection on the pullets fed the basal diet treatment on day 1 and 10, while the main antioxidative parameters SOD, GSH-PX, and T-AOC were lower in the CON group than those of the DIQ group, which supports previous results (17, 18, 36). Similarly, the previous result showed that diquat down-regulated the expression levels of *SOD-1* and *GPX-1* in rats' spleen (37), but Chen et al. found that diquat increased the expression level of *GPX-1* in the liver of broilers (38). These results confirmed that the antioxidative capabilities of pullets were impaired after injection with diquat. This was due to the excessive production of ROS with an increase in the level of MDA, finally, the product of peroxidation of polyunsaturated fatty acids, and related esters, and cells can be protected from oxidative damage with SOD, which converts superoxide anion to hydrogen peroxide that is removed by GSH-Px (36). Resveratrol, a plant polyphenol, appears to have a potent antioxidant property via increasing levels of GSH and modulating antioxidant enzymes (MDA, SOD, and CAT) (24, 39). A study in mice proved that the level of SOD in serum was improved by dietary resveratrol supplementation, whereas the MDA concentration in the serum was downregulated (40). Furthermore, The levels of SOD and GSH-PX in the plasma of sows were increased, meanwhile, the MDA level was decreased by resveratrol supplementation (41). As for the studies on chickens, dietary supplementation of resveratrol was found to protect against heat stress by increasing the muscle GSH-PX and T-AOC activities and decreasing muscle MDA levels (42). Moreover, resveratrol could ameliorate heat stress in chickens by enhancing their antioxidant capacity and reducing their MDA content (34, 43). Previous studies showed that resveratrol increased the expression level of *SOD-1* in tissues to resist oxidative stress and heat stress in rats (44, 45). *In vitro*, the expression level of *SOD-1* was upregulated by resveratrol in cells under H_2_O_2_-induced (46, 47). Apigenin is a flavonoid compound abundantly present in common fruits and vegetables, increasingly noted for its various pharmacological effects, especially its anti-oxidation property (48). Studies about the antioxidant effect of apigenin are mainly based on *in vitro* cellular experiments, and only a few studies have investigated the antioxidant effect of apigenin *in vivo* on chickens. Our results revealed that the activity of several essential enzymes participating in the antioxidant defense system, namely SOD, T-AOC, and GSH-PX, was significantly enhanced by apigenin post-diquat treatment. Conversely, the MDA level was inhibited by apigenin on days 1 and 10, which is in accordance with previous studies (49, 50). Therefore, the improved antioxidative capacity induced by resveratrol and apigenin may partly explain why resveratrol and apigenin are beneficial to the pullets under oxidative stress.

The intestinal epithelial barrier is the first line of defense against a hostile environment within the intestinal lumen. We found that administering a diquat injection to chickens disrupted their intestinal morphology, with large amounts of intestinal villi shed and large quantities of epithelial cells appearing necrotic, findings similar to a few studies (18, 20, 51). Other research works showed that resveratrol treatment partly improved the histological morphology of jejunum in heat-stressed rats (44); a diet supplemented with resveratrol countered the impairment of intestinal morphology in heat-stressed broilers (42); and resveratrol supplementation significantly protected against intestinal morphological damage and weakened intestinal integrity in weaning piglets (52). Thus, these studies suggest that resveratrol supplementation can benefit intestinal health. Similarly, we found that supplementation with resveratrol developed the morphology of jejunum and ileum. Moreover, the present study is the first to explore the ameliorative effect of apigenin supplementation against oxidative stress and intestinal morphological changes induced by diquat in pullets. As expected, administration of apigenin reduced the oxidative stress response induced by diquat and relieved the injury of ileum and jejunal morphology in pullets.

As a pivotal sensor of oxidative stress, nuclear factor erythroid 2-related factor 2 (NRF2) plays a central role in the regulation of antioxidant and phase 2 detoxifying enzymes and related proteins (53). GSH is one of the most versatile cellular antioxidants, and all enzymes involved in GSH biosynthesis are controlled by NRF2 (54). Previous studies have shown that NRF2 can regulate the antioxidant response and represents the underlying mechanism that provides a pivotal defense in animals against diquat toxicity (55). Once stimulated by inducers, NRF2 activates downstream enzymes, including NQO1 and HO-1, to prevent oxidative stress damage from occurring. In this study, the diquat challenge downregulated the intestinal *NRF2* expression and its downstream target genes *NQO1* and *HO-1*. This could be inferred as an acute response to diquat-induced oxidative stress, which was in agreement with the results (56, 57). Resveratrol could not increase the expression of the *NRF2* gene in the liver of rats exposed to high temperature (45), akin to our result that resveratrol was unable to upregulate the expression of the *NRF2* gene, except in ileum on day 10. However, a previous study did show that the NRF2 protein and *HO-1* gene expression levels are increased in pigs by dietary resveratrol supplementation (41). Moreover, a resveratrol treatment considerably increased the HO-1 protein levels in the heart tissue of rats, suggesting resveratrol greatly improved their antioxidant ability (58). *In vitro*, an H_2_O_2_ treatment markedly decreased the expression of *NRF2* and *HO-1* in cells, whereas resveratrol significantly reversed the H_2_O_2_-induced downregulation of *NRF2* and *HO-1* (47, 59). Apigenin has been capable of augmenting the expression of HO-1, NQO1, and GCLM at both the mRNA and protein levels (60). Similarly, apigenin dramatically raised the mRNA and protein expression of NRF2, HO-1, and NQO1 to higher levels in a dose-dependent manner (61). The present study showed that resveratrol and apigenin supplementation for 10 days markedly promoted the mRNA expressions of *HO-1* and *NQO1* in the ileum and jejunum of diquat-induced pullets. These results suggest that resveratrol and apigenin have a time-dependent effect on the anti-oxidative stress of the small intestine involving the Nrf2 pathway caused by diquat.

The intestinal barrier is physically composed of epithelial cells connected by tight junction proteins, such as ZO-1, CLAUDIN-1, and OCCLUDIN, which regulate epithelial cell-selective permeability. Therefore, the expression of genes is crucial for maintaining a functionally intact intestinal epithelial barrier (62). The family of ZO is a part of the cytoplasmic plaque of the tight junction proteins, and the occludin and clauding families are markers of tight junction integrity found in the epithelial barrier; their presence or absence could reflect the permeability of the intestinal epithelium (63). The integrity of the intestinal mucosal barrier prevents and defends against the invasion of stimulating factors and bacteria and further mitigates intestinal inflammation and oxidative stress (64). Tight junction mRNAs and proteins expression can vary according to the exposure to diquat (18, 20), for example, diquat challenge decreased the mRNA and protein levels of OCCLUDIN, CLAUDIN-1, and ZO-1 in the jejunal mucosa of piglets compared with the control group (65), which matched our results. However, we noticed that oxidative stress had negative effects on *CLAUDIN-1* gene expression in the ileum, which is consistent with the results of a previous study (20). Whether this phenomenon is induced by diquat still requires further investigation, and it may be related to a compensatory effect. According to other research, resveratrol could prevent diquat from causing a decline in the *CLAUDIN-1* expression level in jejunal mucosa of piglets (66). However, there was no significant difference in the mRNA expressions of *OCCLUDIN* and *CLAUDIN-1* between the resveratrol groups and the control group in jejunal mucosa of piglets (52). This phenomenon suggests that resveratrol and apigenin supplementation may result in different expression patterns of tight junction genes in the ileum and jejunum. The current results collectively provide essential evidence of the potential protective effects of resveratrol and apigenin against diquat-induced dysfunction in the intestinal barrier.

## Conclusions

Collectively, the results of this study demonstrated that dietary supplementation of resveratrol and apigenin conferred relieved the intestinal oxidative stress by modulating the NRF2 pathway and tight junction mRNAs in diquat-induced pullets, indicating that resveratrol and apigenin could be promising antioxidant additives for use in mitigating oxidative stress and protecting the intestinal barrier of farmed poultry. However, further investigation is needed for a deeper understanding of the action mechanisms of resveratrol and apigenin in diquat-induced animal models.

## Data Availability Statement

The datasets presented in this study can be found in online repositories. The names of the repository/repositories and accession number(s) can be found in the article/supplementary material.

## Ethics Statement

The animal study was reviewed and approved by the Local Experimental Animal Care Committee and all procedures were implemented and approved by Nanjing Agricultural University.

## Author Contributions

NZ conducted the animal experiments and wrote the manuscript. YT, WX, and TG contributed to the study design. WL and BT executed the lab analysis and performed the statistical analysis. KZ and LL revised the paper. All authors carefully read and approved the final revision of the manuscript.

## Funding

This work was supported by Zhejiang Provincial Key Research and Development Plan (No. 2021C02034) and Zhejiang Provincial Special Commissioner Team Projects of Science & Technology (No. Xianju Chicken Industry, 2020–2024).

## Conflict of Interest

WL was employed by Huzhou Lvchang Ecoagriculture Co., Ltd. The remaining authors declare that the research was conducted in the absence of any commercial or financial relationships that could be construed as a potential conflict of interest.

## Publisher's Note

All claims expressed in this article are solely those of the author and do not necessarily represent those of their affiliated organizations, or those of the publisher, the editors and the reviewers. Any product that may be evaluated in this article, or claim that may be made by its manufacturer, is not guaranteed or endorsed by the publisher.
